# Lack of awareness of liver organ damage in patients with type 2 diabetes

**DOI:** 10.1007/s00592-021-01677-y

**Published:** 2021-01-26

**Authors:** Stefano Ciardullo, Tommaso Monti, Gianluca Perseghin

**Affiliations:** 1 Department of Medicine and Rehabilitation, Policlinico Di Monza, Via Modigliani 10, 20900 Monza, MB Italy; 2 Department of Medicine and Surgery, University of Milano Bicocca, Milan, Italy; 3 Department of Statistics and Quantitative Methods, University of Milano Bicocca, Milan, Italy

**Keywords:** NAFLD, MAFLD, Diabetes, Screening, Fibroscan, Cirrhosis, Awareness

## Abstract

**Aims:**

Liver steatosis, a typical finding in patients with type 2 diabetes (T2D), can lead to cirrhosis and hepatocellular carcinoma. The aim of the present study is to estimate the awareness of liver disease among patients with T2D and whether it differs according to the degree of liver fibrosis estimated by transient elastography (TE).

**Methods:**

This is a population-based cross-sectional study. We included all patients with T2D that participated in the 2017–2018 cycle of the National Health and Nutrition Examination Survey and underwent a TE examination. Presence of liver steatosis and fibrosis was assessed by the median values of controlled attenuation parameter and liver stiffness measurement, respectively.

**Results:**

Among the 825 patients included in the analysis, 8.1% (95% CI 5.1%-12.7%) of patients with steatosis were aware of having a liver condition. Even if awareness increased proportionally with increasing severity of organ damage, it remained limited even among patients with advanced fibrosis (17.9%, 95% CI 8.8%-33.3%).

**Conclusions:**

Despite increasing evidence of a frequent hepatic involvement associated with poor prognosis, awareness of suffering of advanced liver disease in patients with T2D is remarkably low, likely reflecting little recognition also among the team of health care professionals.

## Introduction

Patients with type 2 diabetes (T2D) are at higher risk of dying from liver-related conditions compared to the general population. While excess risk may be present for viral and alcohol-related hepatic diseases, most of it is attributable to nonalcoholic fatty liver disease (NAFLD) [1]. NAFLD affects 60–70% of patients with T2D, who are also at higher risk of progression toward nonalcoholic steatohepatitis (NASH), cirrhosis and hepatocellular carcinoma [2, 3].

Nonetheless, due to many uncertainties, including incomplete information on the natural history of the disease, challenges in the diagnosis of NASH, and few pharmacological agents with proven efficacy, previous studies in the general NAFLD population reported low awareness of this condition among affected individuals [4].

Here, we analyzed data from the 2017–2018 cycle of the National Health and Nutrition Examination Survey (NHANES) to estimate the awareness of liver disease among patients with T2D according to the degree of liver fibrosis, estimated by transient elastography (TE).

## Materials and methods

This is an analysis of data from the 2017–2018 cycle of NHANES, a cross-sectional survey program conducted in the US and aimed at including individuals representative of the general, non-institutionalized population of all ages. The survey consists of a structured interview conducted in the home, followed by a standardized health examination that includes a physical examination as well as laboratory tests. The original survey was approved by the Centers for Disease Control and Prevention Research Ethics Review Board, and written informed consent was obtained from all adult participants.

The present study focuses on adult patients (age ≥ 20 years) with T2D and reliable TE results. Diagnosis of T2D was based on a prior self-reported diagnosis of diabetes and/or a Hemoglobin A1c (HbA1c) level ≥ 48 mmol/mol, with the exclusion of patients with probable type 1 diabetes (age at diagnosis < 30 years and insulin as the only anti-hyperglycemic drug) [5]. In the 2017–2018 cycle, TE was performed by NHANES technicians after a 2-day training program with an expert technician, using the FibroScan® model 502 V2 Touch (Echosens, Paris, France). Exams were considered reliable only if at least 10 liver stiffness measurements (LSM) were obtained after a fasting time of at least 3 h, with an interquartile (IQRe) range / median < 30%. Median controlled attenuation parameter (CAP) values ≥ 274 db/m were considered indicative of steatosis, median LSM ≥ 8.2 kPa was considered indicative of significant (≥ F2) fibrosis, whereas values ≥ 9.7 kPa were considered indicative of F3–F4 [6].

A patient was considered aware of a liver condition if he answered “yes” to the following question, which was part of the medical conditions questionnaire: “Has a doctor or other health professional ever told you that you had any kind of liver condition?” Furthermore, if the patient answered positively to the previous question, information was gathered on the type of liver disease, which was classified as follows: “fatty liver”, “liver fibrosis”, “liver cirrhosis”, “viral hepatitis”, “autoimmune hepatitis” and “other liver disease”.

Viral hepatitis was also assessed by measuring Hepatitis C RNA and confirmed antibodies and hepatitis B surface antigen. Laboratory methods for measurements of HbA1c, glucose, lipid profile, alanine aminotransferase (ALT), aspartate aminotransferase (AST), γ-glutamyltranspeptidase (GGT), platelet count and albumin are reported in detail elsewhere [7].

All analyses were conducted using SAS version 9.4 (SAS Institute Inc., Cary, North Carolina), accounting for the complex survey design of NHANES. We used appropriate weighting for each analysis, as suggested by the NCHS. Data are expressed as numbers and weighted proportions for categorical variables and as weighted means ± Standard Error (SE) for continuous variables. Awareness of liver disease across degrees of liver fibrosis was compared using the design-adjusted Rao-Scott chi-square test. A two-tailed value of *p* < 0.05 was considered statistically significant.

## Results

A total of 825 patients (52.9% men, mean age 60.6 ± 1.05 years, mean body mass index 33.3 ± 0.53 kg/m^2^) were included in the analysis and their clinical and metabolic features are shown in Table 1. A total of 557 patients had evidence of steatosis (weighted prevalence 73.8%, 95% CI 68.5%–78.5%) and 119 had evidence of advanced (F3–F4) fibrosis (weighted prevalence 15.4%, 95% CI 12.2%-19.0%). AST, ALT and GGT levels increased progressively going from patients with F0–F1 (20.1 IU/L, 22.2 IU/L and 30.6 IU/L) to those with F2 (26.7 IU/L, 34.3 IU/L and 44.9 IU/L) and F3-F4 (30.2 IU/L, 32.7 IU/L and 64 IU/L, *p* < 0.01 for all).

**Table 1:** Clinical and metabolic features of the studied population

Characteristic	Entire population (*n* = 825)
Male participants (%)	52.9 ± 3.26
Age (years)	60.6 ± 1.05
Diabetes duration (years)	10.1 ± 1.03
BMI (Kg/m^2^)	33.3 ± 0.53
Obesity (%)	64.1 ± 2.96
Waist circumference (cm)	111.9 ± 1.05
Current smoke (%)	27.0 ± 4.02
Ethnicity (%)	
Non-Hispanic white	56.9 ± 3.30
Non-Hispanic black	13.3 ± 2.44
Hispanics	16.1 ± 1.94
Asian	7.5 ± 1.24
Others	6.2 ± 1.10
Hepatitis B (%)	1.00 ± 0.03
Hepatitis C (%)	0.4 ± 0.21
Laboratory features	
HbA1c (%, mmol/mol)	7.2, 55.2 ± 0.46
AST (U/l)	22.2 ± 0.57
ALT (U/l)	24.8 ± 0.91
GGT (U/l)	36.9 ± 1.99
Platelet count (× 10^9^/L)	241.9 ± 5.44
Albumin (g/l)	39 ± 0.2
Total Cholesterol (mmol/l)	4.7 ± 0.07
HDL-cholesterol (mmol/l)	1.2 ± 0.02
Triglycerides (mmol/l)	2.3 ± 0.13
LDL-cholesterol (mmol/l)	2.4 ± 0.06

Data are expressed as weighted proportions (± Standard Error (SE)) for categorical variables and as weighted means ± SE for continuous variables

*BMI* Body mass index; *HbA1c*, Hemoglobin A1c; *AST*, Aspartate aminotransferase; *ALT*, Alanine aminotransferase; *GGT*, Gamma-glutamyltranspeptidase; *HDL*, High density lipoprotein; *LDL*, Low density lipoprotein

**Fig. 1:**
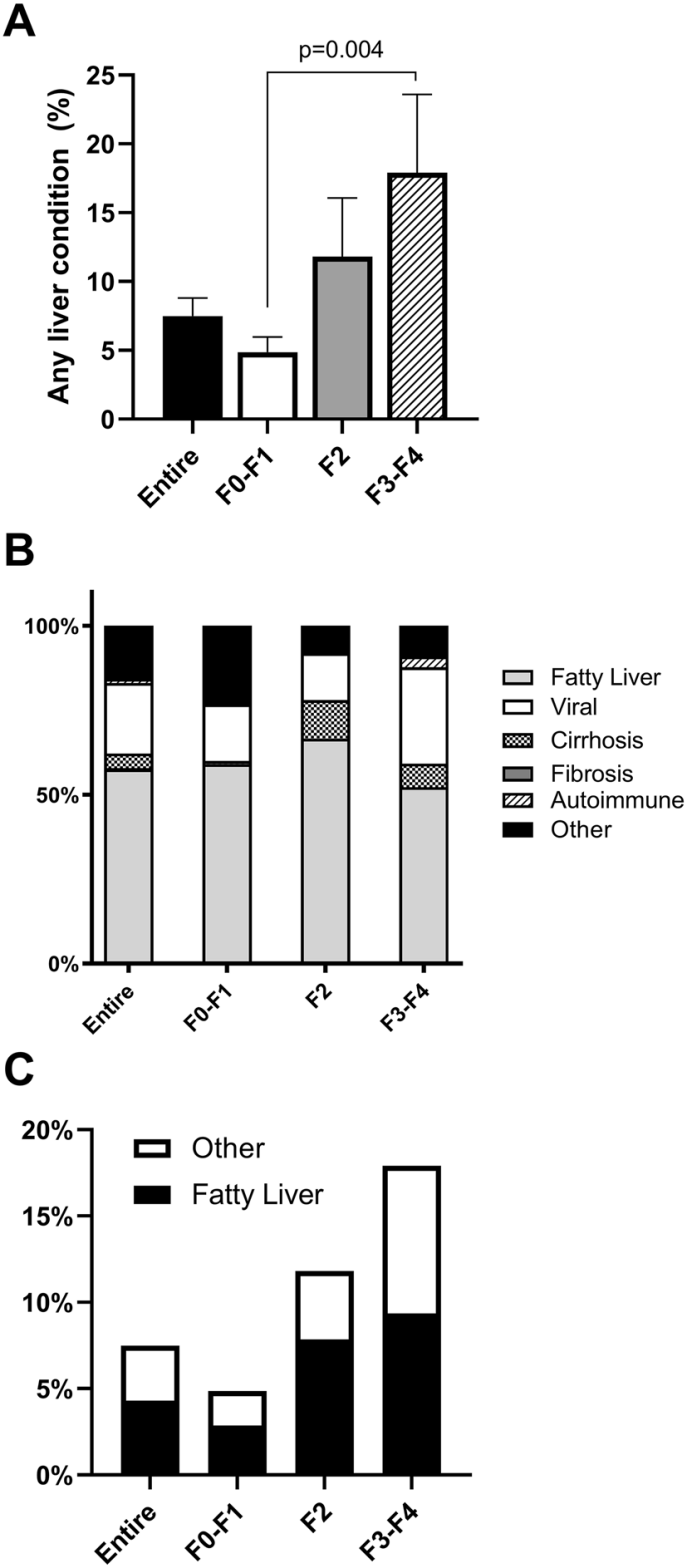
Liver disease in the studied population. Proportion of patients aware of having any kind of liver condition according to the estimated degree of liver fibrosis (**a**) and type of liver disease among aware patients (**b**, **c**). NHANES, National Health and Nutrition Examination Survey

Awareness of any liver condition was 7.5% (95% CI 5.1%–10.8%) in the entire population and not significantly different at 8.1% (95% CI 5.1%–12.7%) in patients with steatosis. Furthermore, it increased going from patients with F0–F1 (4.9%, 95% CI 2.9%–7.8%) to those with F2 (11.8%, 95% CI 5.3%–24.3%) and F3–F4 (17.9%, 95% CI 8.8%–33.3%), as shown in Fig. 1, panel a. The difference was significant between patients in the F3–F4 and in the F0–F1 group (*p* = 0.004), but not between F0–F1 and F2 or between F2 and F3–F4. No significant differences were found in the reported type of liver disease, with fatty liver being the most common condition in all groups (52.2%–66.5%), followed by viral hepatitis (13.9%–28.5%) and “other” causes, which can be mainly attributed to alcohol (Fig. 1, Panels b and c).

## Discussion

To our knowledge, this is the first study highlighting the low awareness of having a liver condition in a representative population of US adults with T2D. We show that, although awareness increases with increasing severity of liver disease, it is lower than 20% even in patients with elastographic evidence of advanced fibrosis.

In a previous study performed in the general US population, Singh et al. found that awareness of liver disease was extremely low among individuals with suspected NAFLD (defined as a BMI ≥ 25 kg/m^2^ and elevated ALT levels), with an increase from 1.5% to 3.1% from 2001–2004 to 2013–2016 [8]. Importantly, awareness was also low in patients with advanced fibrosis estimated through non-invasive scores. Similarly, in a study from Colorado, among subjects at high metabolic risk attending an endocrinology clinic of a single academic hospital, only 18% were aware of NAFLD as a disease entity [9].

Our estimates expand data from the existing literature by focusing exclusively on patients with T2D and evaluating whether the degree of liver fibrosis assessed through a well-performing and validated non-invasive technique impacts on the level of awareness. It should also be considered that mean diabetes duration in our study was ~ 10 years and that the prevalence of advanced liver fibrosis might be higher in populations with a longer diabetes history. Several explanations can be advanced to explain this lack of awareness.

First, lack of awareness on prevalence, diagnosis and guidelines for NAFLD has been shown among primary care physicians and non-hepatologist hospital specialists. In a study from Queensland, 51% of primary care physicians believed the prevalence of NAFLD to be lower than 10% in the general population and 70.6% said they were unlikely to refer a patient to hepatology unless liver function tests were abnormal [10]. Similarly, 71% of hospital specialists from the same area make no referrals to hepatology for suspected NAFLD [11]. Second, chronic liver disease and NAFLD in particular do not lead to symptoms until decompensated cirrhosis or hepatocellular carcinoma develop, which occurs in a low number of subjects.

Third, as a consequence of a lack of evidence on cost-effectiveness of NAFLD screening and uncertainties on how to perform it, guidance from international societies has been heterogeneous. While guidelines from the European Association for the Study of the Liver (EASL), Diabetes (EASD) and Obesity (EASO) recommend routine screening for NAFLD in patients with T2D, independently from liver enzymes, the American Association for the Study of Liver Diseases does not endorse it, but encourages case finding if suspicion of NASH is high [12].

Our study has the advantage of a relatively large sample of patients with T2D and a high level of generalizability to the US population. Moreover, it is, to our knowledge, the first study to report awareness across the spectrum of liver fibrosis assessed by transient elastography, which is among the best performing and most validated non-invasive techniques to detect liver fibrosis. Nonetheless, several limitations should be acknowledged. First, lack of liver biopsy data does not allow to report the prevalence of steatohepatitis and different degrees of fibrosis. Second, data on alcohol consumption are not available, preventing us from quantifying the exact contribution of NAFLD to the measured prevalence of advanced fibrosis. On the other hand, LSM cutoffs to identify F3–F4 fibrosis are similar in different liver conditions. Therefore, irrespective of the specific etiology, we show that few patients with advanced liver fibrosis are aware of their condition.

## Conclusions

In conclusion, in a nationally representative sample of US adults with T2DM, prevalence of advanced liver fibrosis is high. Nonetheless, less than 20% of those with advanced fibrosis are aware of having any kind of liver condition.

## Data Availability

All data used in this study are publicly available online at the NHANES website.

## Abbreviations

T2D: Type 2 diabetes
NAFLD: Nonalcoholic fatty liver disease
NASH: Nonalcoholic steatohepatitis
TE: Transient elastography
LSM: Liver stiffness measurement
CAP: Controlled attenuation parameter
NHANES: National health and nutrition examination survey
